# New Scenarios in Pharmacological Treatments of Head and Neck Squamous Cell Carcinomas

**DOI:** 10.3390/cancers13215515

**Published:** 2021-11-03

**Authors:** Cristina Porcheri, Thimios A. Mitsiadis

**Affiliations:** Institute of Oral Biology, Faculty of Medicine, University of Zurich, Plattenstrasse 11, CH-8032 Zurich, Switzerland; thimios.mitsiadis@zzm.uzh.ch

**Keywords:** head and neck squamous cell carcinoma, oral cancer, drug testing, drug delivery, organoids, spheroids, exosomes

## Abstract

**Simple Summary:**

Head and neck squamous cell carcinoma (HNSCC) encompasses a variety of tumors developing in the head and neck region, mainly affecting the oral cavity. The past decades have seen little advancement in the understanding of the biological basis of HNSCC, which strongly hampered the development of novel, more effective treatments. Here, we summarize the current therapies available for the treatment of HNSCC and give an overview of new models for screening and testing emerging therapeutic approaches.

**Abstract:**

Head and neck squamous cell carcinoma (HNSCC) is one of the most frequent types of cancer with a lethal outcome in half of the diagnosed cases. Mostly, HNSCC develops in the oral cavity, and its development is associated with tobacco and areca nut/betel quid usage, alcohol consumption, and HPV infection. Oral squamous cell carcinoma, as other head and neck cancers, presents a high degree of intratumor heterogeneity, which makes their treatment difficult, and directly correlates with drug resistance. Since the classical treatments for HNSCC oftentimes do not resolve the clinical picture, there is great need for novel therapeutic approaches, models for drug testing, and new drug delivery systems.

## 1. Introduction

### 1.1. Squamous Cell Carcinoma in the Head and Neck Region

#### 1.1.1. Distribution and Incidence

Head and neck squamous cell carcinoma (HNSCC) represents the sixth most common cancer worldwide with 880,000 new cases registered in 2018 [1]. It mainly affects male patients and has a lethal outcome in 51% of the cases [1,2,3]. HNSCC originates in many anatomical regions, such as the larynx, hypopharynx, oropharynx, nasopharynx, and oral cavity. According to the cancer size and its depth of invasion, HNSCC is classified as T1, T2, T3, and T4 types, with T1 being the smallest and least aggressive and T4 the most aggressive type (moderately advanced or very advanced disease) [1,2,3]. In total, 60% of HNSCC belong to the oral squamous cell carcinoma (OSCC), which develops at the alveolar ridge, buccal mucosa, floor of the mouth, hard palate, lip, and tongue [4,5].

#### 1.1.2. Causes

**Figure 1:**
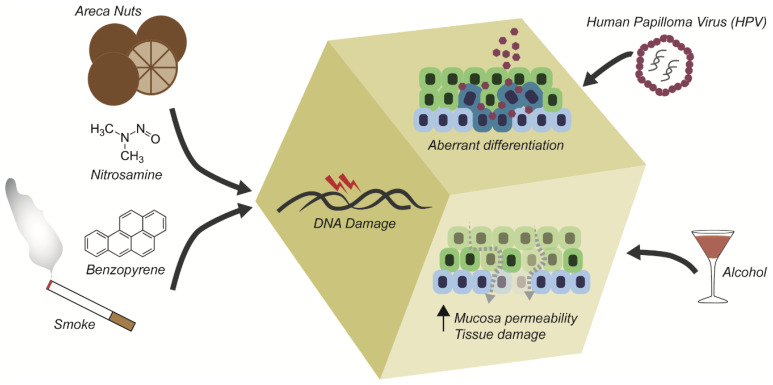
Cancerogenic molecules and way of action. HNSCC strongly correlates with usage of areca nuts, tobacco, alcohol consumption, and HPV infection. Active molecules have direct effects on tissue structure, ultimately altering the barrier capability of the epithelium and increasing cellular damage.

The most common causes of OSCC are tobacco and areca nut/betel quid usage, alcohol consumption, and HPV infection [6,7,8] (Figure 1). The risk for developing oral cancer is 5 to 25 times higher in smokers compared with non-smokers, also in relation to the quantity and duration of exposure to carcinogens [6,9,10]. Cigarette smoke contains a plethora of pre-carcinogenic molecules, belonging mainly to three different families: benzopyrenes, nitrosamines, and aromatic amines. These molecules face alterations mediated by oxidative enzymes, become reactive metabolites, and promote the emergence of mutations upon physical interaction with DNA [6,7,11,12,13]. Chronic usage of tobacco and alcohol has a synergistic effect in disrupting the oral mucosa structure, causing epithelial lesions [9,14]. Alcohol causes oral epithelial atrophy by interfering with the lipid’s composition of the epithelial layer, hence leading to damage in the DNA synthesis and repair processes [6,15,16].

After tobacco and alcohol, the main cause of OSCC is the consumption of the areca nut. The consumption of the nut is a cultural habit in South Asian countries and directly correlates with the development and high frequency of OSCC [17]. The nut can be chewed alone, or in a mixture with other substance (including tobacco), receiving the name of betel quid [18]. The areca nut itself has a very diverse composition, containing polyphenols, tannins, and alkaloids, with the arecoline (alkaloid) being the main carcinogen [19]. Arecoline is transformed into nitrosamines that interact with DNA via chromatin relaxation [16,20,21,22,23,24,25,26].

Another main cause of oral cancer is the infection from human papillomavirus (HPV). HPV16 and HPV18 infections together are the cause of 40% of all OSCC, usually developing at the oropharynx and base of the tongue. In general, patients with OSCC caused by HPV present a better prognostic than HPV-negative patients with OSCC. This could be directly correlated with the carriage of the viral protein E6 that inactivates TP53, one of the most mutated genes in OSCC [5,6,27,28,29].

#### 1.1.3. Intra-Tumor Heterogeneity (ITH)

A few studies have suggested that malignant cells have the ability to recruit cells from the surrounding tissues, supporting tumor invasion, progression, proliferation, and metastases [30]. The newly formed tumor microenvironment (TME) allows the tumor to thrive, as it protects, nourishes, and sustains cancer growth. Several elements are part of the TME: immune system components (e.g., T-cells, B/plasma cells, macrophages, dendritic cells, and mast cells), endothelial cells, fibroblasts (e.g., myofibroblasts and CAFs), myocytes, and malignant cells themselves [31]. The interaction between malignant cells and the TME is essential to maintain tumor homeostasis and survival [32,33], although the details of how this communication exactly occurs remain to be clarified. Malignant cells can manipulate their surroundings to their advantage, influencing, among others, hypoxia, cell cycle, and differentiation processes [31]. Novel sequencing techniques, such as next-generation sequencing techniques (NGS), allow for the identification of genetic variability and a general understanding of intra-tumor heterogeneity (ITH) [34,35,36]. It has been demonstrated that not only is ITH involved in drug resistance [35,37], but also that the order in which the mutations appear can influence the clinical evolution of the malignancy [35,38]. Recurrence and metastatic behavior processes are often occurring in OSCC, with the appearance of lymph node metastasis in the majority of cases [39,40]. As the presence of metastases indicates the capability of tumor cells to escape from the original site and build a supportive microenvironment elsewhere, it directly correlates with their ability to elude internal surveillance and circumvent therapy. Therefore, the appearance of lymph node metastasis directly correlates with therapy resistance and likely contributes to the lack of improvement in the 5-year survival rate registered worldwide over the last few years [41,42].

## 2. Therapeutic Approaches for Head and Neck Squamous Cell Carcinoma

### 2.1. Current Therapies

**Figure 2:**
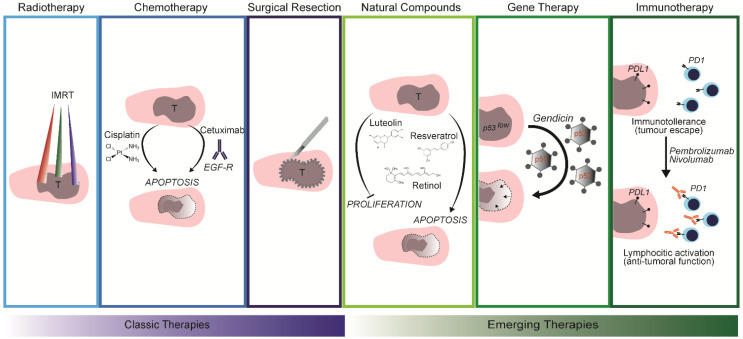
Comparison between classic therapeutic approaches and emerging therapies. Radiotherapy, chemotherapy, and surgical resection are the most common interventions on HNSCC. Novel therapeutic approaches that exploit the anti-cancer properties of natural compounds, viral-vector mediated gene therapy, and immunotherapy are emerging.

Classical treatments of HNSCC are mainly based on surgical resection followed by radiotherapy and chemotherapy, with specificity of treatment depending on different factors (e.g., pre-existing clinical conditions and location and stage of the tumor) [5,7,43,44]. Radiotherapy techniques applied to HNSCC patients are often 3D conformal radiotherapy and intensity-modulated radiotherapy (IMRT) (Figure 2). The latter is considered to be more precise, reducing the damage caused by irradiation in the surrounding healthy tissue [45]. Amongst the chemotherapeutic agents, the most common for HNSCC treatment is cisplatin, a platin-based compound that binds the purine components of DNA, forming adducts and inducing apoptosis [46]. However, since cisplatin alone is often not efficient, other drugs have been used upon genetic screening for patient-specific mutations. Most HNSCC patients present mutations in the epidermal growth factor receptor (EGFR). In these cases, cetuximab, a monoclonal antibody against EGFR, has been efficiently used either alone or in combination with radiotherapy or chemotherapy [5,47,48].

### 2.2. Emerging Therapies

Most of the time, the traditional treatments for HNSCC do not resolve the clinical outcome, and, therefore, innovative therapeutic approaches have started to be applied, such as the usage of natural compounds, gene therapy, and immunotherapy (Figure 2).

Natural compounds, such as vitamin A, luteolin, and resveratrol, have shown therapeutic potential in treating HNSCC [49,50,51]. Their mechanism of action involves induction of apoptosis and a consequent decrease in tumor size whilst facing important limitations (e.g., poor bioavailability and dose-dependent toxicity) [52,53,54,55,56,57,58,59].

Many different genetic mutations found in HNSCC patients assist in tumor cell survival, invasiveness, and therapy resistance. Amid the most common altered genes, *p53* has a high mutation frequency [60,61]. Gendicine, the first gene therapy approved for HNSCC, is based on an antitumor effect by restoring p53 function using an adenoviral vector delivery system. Instead of killing the tumor, the adenovirus stimulates the tumor cells to express the corrected form of p53 and consequently restore its normal function [62]. Cytoreductive gene therapy has also been tested for HNSCC. Transgene-mediated, tumor-specific activation of the prodrug induces the expression of its toxic metabolite. This approach is known as “suicide gene therapy”, as once the prodrug is internalized in the cancer cells, its toxic metabolite leads to programmed cell death [60,63].

Immunotherapy is a novel promising therapy based on the exploitation of the immune system’s potential to fight cancer. Pembrolizumab and nivolumab are the two drugs approved for immunotherapy in HNSCC patients. They are antibodies anti-PD1, a protein that regulates immune response. They are currently being used in patients presenting with recurrent or metastatic HNSCC [5,64,65,66,67].

## 3. Modeling HNSCC for Drug Testing

Two-dimensional (2D) cell culture systems are a common in vitro model to study cell biology and reaction to treatment. They allow rapid acquisition of results, have low maintenance costs, and require minimal establishment by the experimenter. Traditional drug testing performed in 2D cultures allowed us to have a better understanding of the potential effects and toxicity of the new drugs. However, 2D cultures only partially replicate the complex environment found in patients, and, thus, the use of more appropriate culture model systems is essential for drug testing [68,69,70].

Three-dimensional (3D) culture models (e.g., organotypic cultures, spheroids, and organoids) are more similar to the native tumor regarding cell heterogeneity, genetic variability, and cell to microenvironment interaction, thus representing an excellent platform for personalized medicine and drug screening [68,71].

Amongst the various subtypes of 3D models, spheroids are the simplest model. In general, spheroids contain a proliferating cell layer (external layer) and a quiescent cell layer (internal layer) [71,72,73,74,75,76,77,78,79,80,81,82,83,84,85,86]. Various techniques can be used to grow and maintain spheroids in culture, with applications ranging from studies on tumor microenvironment to drug screening and molecular testing [68,78,87,88]. In one of the most common methods for spheroid production, the hanging drop, a drop of culture medium containing patient’s derived cells, is plated, suspended, and cells aggregate by gravity at the bottom of the drop. The popularity of this technique is mainly associated with the low cost and uniform size of the spheroids obtained, although their survival in culture conditions is limited [89]. The liquid overlay technique consists of generating spheroids using non-adherent surfaces (e.g., ultra-low attachment plates). The spheroid is developed in an individual well and can be accessed easily for treatment and manipulation. On the other hand, the size of the spheroid will depend on the size of the well, which might influence drug permeability within the spheroid [89,90]. Finally, the scaffold-based 3D cell culture relies on the presence of a specific matrix (e.g., Matrigel or polyethylene glycol (PEG)). Matrigel is generated from mouse sarcoma cells and contains factors that are present in native tumors, adequately mimicking the drug response in the presence of a complex tumor microenvironment. The components of the matrix stimulate cell growth and proliferation, two necessary processes contributing to spheroids generation and development. Rich matrices (e.g., Matrigel) act as reservoirs of growth factors that actively sustain a spheroid’s growth, while simpler synthetic matrices (e.g., PEG) provide only a basic structural support. Additionally, synthetic matrices might limit drug penetrability and potentially alter drug responses [15,88,91,92,93,94,95,96,97]. The use of spheroids for drug testing in HNSCC is still in its developmental phase, mainly due to the high intrinsic variability of the tumor and the lack of consensus in the technology for spheroid production. A comparison between 2D and 3D culture effectiveness of therapy highlights how spheroids display mechanisms of resistance to treatment not observed in monolayer cell cultures [71,98,99,100]. On the other hand, important limitations still exist in using spheroids for drug testing. Besides the complexity in establishment and manipulation, the size of spheroids can largely vary, influencing drug penetrance, efficiency, and, ultimately, reproducibility of results [71,85].

Organoids can also be used to study TME and drug screening [101,102]. They can be cultured in a matrix, or in suspension, with specific supplemented media [68,101,103]. Organoids can be expanded from patient-derived cells and maintained in culture for long periods, allowing the development of personalized biobanks [68]. Cancer-derived organoids are able to preserve the characteristics of the original tumor [101,104,105,106], including tumor metabolism [68,107], and, therefore, constitute ideal tools for identifying novel cancer-specific biomarkers [108,109]. Cryopreserved patient-derived organoids can be used in the future for personalized drug screening and evaluating individual toxicity and efficacy [101]. Although cancer-derived organoids have been generated in the last few decades from a vast variety of cancer types, HNSCC-derived organoid formation is quite recent. The first OSCC organoids were generated in 2018 from either cancer cell lines or primary cells from oral cancers [68,109,110]. Tanaka and colleagues compared the effects of cisplatin and docetaxel in 2D cultures and in organoids, and demonstrated different sensitivities to drug treatment, with organoids being more resistant to docetaxel [108]. The fact that organoids can be established from tumor tissues and grow rapidly supports the idea of using organoids to study the best therapeutic approach for any individual patient. OSCC-derived organoids can be used to test the efficacy of current chemotherapeutic treatment, demonstrating that the monoclonal antibody cetuximab did not present a radio-sensitizing effect in organoids derived from HNSCC. Some tests face the limitations of the organoid model itself, such as the presence of common genetic mutations. For instance, many of the organoid’s lines used present mutation in PIK3CA and, therefore, cannot be used to predict drug response for the PIK3CA inhibitor [111]. Additionally, specific protocols of cell maintenance and expansion should be established by the user, and some cell types hardly grow in these conditions (e.g., multilayer epithelium) [112,113]. It is important to mention that it is still not known what the level of cell heterogeneity kept on the various organoids is, an important element in OSCC maintenance [68,114]. Finally, although organoids grow fast and can be cryopreserved, the cost of keeping organoids in culture remains high and inadequate for usage on a large scale [68,106].

**Figure 3:**
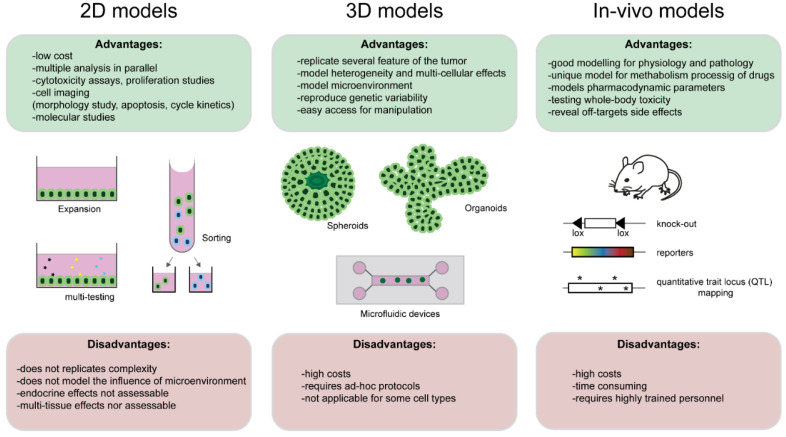
Models for drug screening (in vivo, 2D, 3D comparison). Different types of system modeling for drug screening and pre-clinical drug testing. Two-dimensional cell culture (**left panel**) allows for rapid testing of cytotoxicity, morphological analysis, and basic molecular studies. Three-dimensional culture (**middle panel**) includes in the system the complexity of the microenvironment and intercellular communication. In vivo systems (**right panel**) allow for the study of complex pharmacological interaction occurring in the full organism, including inter-tissue and inter-organ effects.

Animal tests remain the most appropriate for studying and mimicking the physiological and pathological complexity of tumors, but they carry numerous drawbacks (e.g., elevated cost, time consuming, and the need for highly educated personnel) [99]. The actual presence of various transgenic mouse lines allowed us to analyze the impact of systematic medications, with the possibility of following their effect on a specific molecular pathway, either via colored reporter line, functional knock-out or by analyzing genomic complexity via quantitative trait locus (QTL) (Figure 3).

## 4. Novel Drug Delivery Systems

New drug delivery systems can be put in place to improve the pharmacodynamics, efficiency, and sensitivity of novel treatments. Nanoparticles, liposomes, micelles, and exosomes are novel delivery systems currently used to improve the efficiency and safety of drugs.

Nanoparticles can specifically target tumor cells, increasing the bioavailability of drugs and reducing drug dosage, consequently diminishing the side-effects and off-target toxicity [115,116,117,118,119,120,121,122,123,124,125,126,127,128]. The size of nanoparticles can vary between 3 and 200 nm. Nanoparticles developed using colloidal structures composed by lipids generate liposomes, which can be easily modified by adding structural adjustments [129,130]. In polymer-derived nanoparticles, drugs are added to a polymer chain, resulting in a water-soluble compound with high penetrance [129,131,132]. Instead, water-insoluble drugs are hosted in micelles formed by polyethylene glycol (PEG), with an outer hydrophilic surface and a hydrophobic internal core carrying the active agent [132,133,134]. Nanoparticles have been used to improve the treatment of HNSCC. It has been demonstrated that gold nanoparticles covered with cisplatin were able to deliver the drug specifically to HNSCC, also presenting a radio-sensitizing effect [135]. Natural compounds, such as luteolin and resveratrol, have shown great therapeutic potential when delivered via nanoparticles in HNSCC, resulting in tumor growth inhibition [52,115,136,137,138,139]. Despite the fact that HNSCC treatment using nanoparticles looks very promising, there are important limitations, such as an insufficient tissue distribution of nanoparticles, toxicity of some reagents used for nanoparticle generation, and diminished oral bioavailability [129].

**Figure 4:**
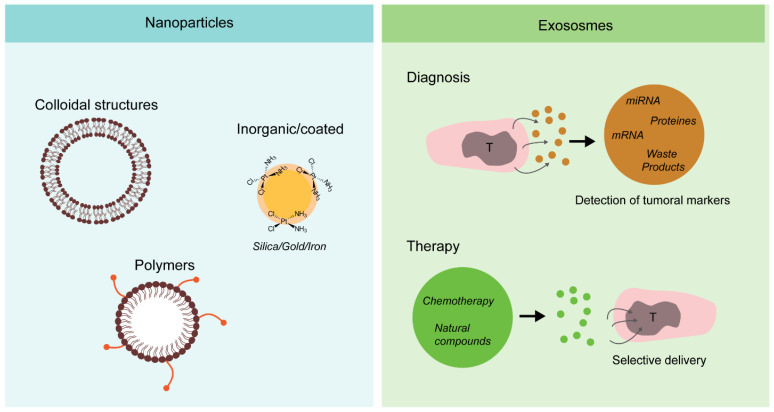
Different delivery routes (classic chemotherapy, nanoparticles, and exosomes). Drug-carried nanoparticles and exosomes can be used for efficient delivery of classical and novel drugs, limiting side effects, and improving availability. Additionally, exosomes released from tumoral cells can be exploited as diagnostic tools and for marker detection.

Exosomes are vesicles released in the extracellular compartment (e.g., microvesicles and apoptotic bodies) [140,141,142,143,144]. They can be found in many of the body fluids, and their sizes vary between 30 and 150 nm [143,144,145,146]. While their role was initially thought to solely participate in cell’s endogenous waste clearance, several observations indicate that exosomes have a much broader function, such as participating in cell signaling, modulating the immune system, and regulating gene expression and intercellular communication [140,141,143,147]. In HNSCC, tumor-derived exosomes have been linked with all stages of cancer development. Exosomes from tumors may participate in cancer initiation, progression and invasion, immune response regulation, and, finally, treatment resistance [144,148,149]. HNSCC patients have a higher quantity of exosomes in plasma, and the number of exosomes increases according to the tumor stage, showing that they can be used as a potential source of HNSCC biomarkers [140,144,150,151,152,153]. HNSCC-derived exosomes contain a variety of microRNAs and factors able to regulate the TME. Particularly, exosomes from HNSCC contain miR-21, a microRNA present in hypoxic cells that are able to promote cell migration and invasion, ultimately stimulating epithelial–mesenchymal transition [154]. They can also regulate immune cells by inhibiting T cells, thus increasing tumorigenesis [144,155,156,157,158]. Additional to their role in pathogenesis, exosomes can be exploited for therapeutic usage. They can be modified and used as exogenous and/or endogenous carriers for drug delivery, increasing their sensitivity to the target tissue [140,159] (Figure 4). However, the safety of exosomes for clinical applications is still under debate, and more studies are needed to grant their routine therapeutic usage in clinics.

## 5. Conclusions

Our knowledge on the biological basis of HNSCC is slowly progressing. This lag of basic knowledge is reflected in reduced possibilities for the development of novel therapeutic strategies. New approaches to drug development include innovative experimental approaches, modeling systems for drug screening, personalized medicine tools, identification of drug targets, and more efficient delivery routes. Advances in these fields of study will pave the way for more efficient and safe therapies to treat HNSCC.
